# Role of BMP-7 on biological parameters osseointegration of dental implants: Preliminary results of a preclinical study

**DOI:** 10.3389/fbioe.2023.1153631

**Published:** 2023-02-28

**Authors:** Nansi López-Valverde, Javier Aragoneses, Antonio López-Valverde, Cinthia Rodríguez, Juan Manuel Aragoneses

**Affiliations:** ^1^ Department of Medicine and Medical Specialties, Faculty of Health Sciences, Universidad Alcalá de Henares, Madrid, Spain; ^2^ Department of Surgery, Instituto de Investigación Biomédica de Salamanca (IBSAL), University of Salamanca, Salamanca, Spain; ^3^ Department of Dentistry, Universidad Federico Henríquez y Carvajal, Santo Domingo, Dominican Republic; ^4^ Faculty of Dentistry, Universidad Alfonso X El Sabio, Madrid, Spain

**Keywords:** dental implants, biological parameters, BMP-7, osseointegration, minipig model

## Abstract

The aim of this work was to analyze and compare the effect of bone morphogenetic protein-7 on biological parameters related to implant osseointegration in an experimental animal model. Sixteen dental implants were placed in the tibias of four randomly selected minipigs for the following dental implant surface treatments: Group A: conventional treatment of the dental implant surface by SLA (*n* = 8) and Group B: treatment of the dental implant surface with carboxyethylphosphonic acid and bone morphogenetic protein-7 (*n* = 8). The animals were sacrificed one month after dental implants placement and a histomorphometric study was performed for the evaluation of bone-to-implant contact, corrected bone-to-implant contact, new bone formation, interthread bone density and peri-implant density using Student’s t-test and the non-parametric Mann-Whitney test. The histomorphometric parameters bone-to-implant contact and corrected bone-to-implant contact showed statistically significant differences between the study groups; 34.00% ± 9.92% and 50.02% ± 10.94%, respectively (*p* = 0.004) for SLA and 43.08% ± 10.76% and 63.30% ± 11.30%, respectively (*p* = 0.003) for BMP-7. The parameters new bone formation, interthread bone density and peri-implant density did not show statistically significant differences between the study groups (*p* = 0.951, *p* = 0.967 and *p* = 0.894, respectively). Dental implant surfaces treated with carboxyethylphosphonic acid and BMP-7 improve the biological response of dental implants to osseointegration.

## 1 Introduction

Currently, dental implant placement is considered a predictable treatment option to restore partially or fully edentulous patients; however, despite high success rates, early failures occur, usually attributable to insufficient osseointegration in the early stages of osseointegration, surgical trauma, and infections (Esposito et al., 1998; Sakka et al., 2012; Alghamdi and Jansen, 2020).

In recent years, research has focused on surface treatment procedures for titanium (Ti) alloy dental implants to enhance their biological response during the osseointegration process and prevent the development of peri-implant diseases (Ferraris et al., 2011). Titanium-aluminum-vanadium alloy (Ti-6Al-4V), in addition to having superior strength, provides biocompatibility and mechanical properties to dental implants (Kaur and Singh, 2019) and this, together with surface treatment techniques, modifying the roughness, topography, chemistry and electrical charge of dental implants, would promote an increase in the biological response of the surrounding peri-implant tissues, and of the microscopic contact surface, which would improve the histological reaction at the bone-implant interface (Gittens et al., 2014; Jemat et al., 2015).

Therefore, manufacturers have subjected dental implants to different technical approaches, based on etching, blasting or a combination of both (Khang et al., 2001; Fini et al., 2003; Szmukler-Moncler et al., 2004; Le Guéhennec et al., 2007; Wennerberg and Albrektsson, 2009; Leon-Ramos et al., 2019) with the aim of increasing the surface roughness and, consequently, the success rate of osseointegration. In particular, it has been described that the application of carboxyethylphosphonic acid (CEPA), enhances the osseointegration process, improving protein adhesion, after generating a physicochemical layer of Al_2_O_3_ or TiO_2_ (Aragoneses et al., 2021). On the other hand, conventional surface treatments (Sandblasted Large-Grit Acid-Etched, SLA type) have shown enhanced osseointegrative power when combined with surface bioactivation, based on the immobilization of proteins, enzymes or peptides, which could induce a specific cellular response in peri-implant tissues in the early stages of osseointegration (López-Valverde et al., 2020). Moreover, in order to promote histological performance, some organic substrates have been used to induce bioactivation; in particular, the osteogenic capacity of bone morphogenetic proteins has been demonstrated “*in vivo*” to induce ectopic bone formation by stimulating bone remodeling (Kim et al., 2017). The latest advances are focused on bioactive surfaces, which endow the mechanical properties of implants with osteoinductive properties, by functionalizing the surface, providing a synergistic effect on osteogenesis through molecular recognition events, determining, in a short period of time, the type of tissue that will develop at the bone-implant interface (Rupp et al., 2018).

Bone morphogenetic proteins (BMPs) are osteoinductive proteins belonging to the transforming growth factor beta (TGF-β) family with the ability to stimulate the differentiation of pluripotential cells towards different cell lineages and promote osseointegration of dental implants (López-Valverde et al., 2022).

Silanization has long been considered, the gold standard coating method for adding organic components to Ti oxide; however, siloxane products can be hydrolytically unstable, which may result in a decrease in the concentration of organic components on the implant surface when the silanized surface is exposed to an aqueous medium (Matinlinna et al., 2013). For this reason, new modifying coatings for implant surfaces have been proposed, through the application of organic acids such as phosphates or carbonates, which, in addition to interacting strongly with Ti oxide, allow the formation of stable surfaces, which bind biomolecules, such as BMPs on the metal oxide, creating a true chemical bond between bone tissue and the implant surface, with absence of fibrous tissue at the bone-implant interface (Liu et al., 2004; Hall et al., 2007; Liu et al., 2007).

The aim of our work was to analyze and compare the effect of BMP-7 on biological parameters related to implant osseointegration in an experimental animal model, with a null hypothesis (H_0_) stating that there were no differences in these biological parameters, between dental implants treated and not treated superficially with BMP-7.

## 2 Materials and methods

### 2.1 Study design

Four (*n* = 4) female minipigs of the Landrace Large White race of 25 kg of weight were selected for this study, according to the European Committee for Standardization guidelines for bone tissue testing. Each animal randomly (Epidat 4.1, Galicia, Spain) received two (*n* = 2) dental implants (grade IV titanium (90 Ti, 6 Al and 4 wt%) 4.0 mm diameter and 10 mm length with an internal taper and conical wall connection (Surgimplant IPX, Galimplant, Sarria, Lugo, Spain) randomly selected (Epidat 4.1, Galicia, Spain) of the following dental implant surface treatments: Group A: conventional dental implant surface treatment through SLA (Galimplant, Sarria, Lugo, Spain) (*n* = 8) and Group B: dental implant surface treatment with CEPA and BMP-7 (Galimplant, Sarria, Lugo, Spain) (*n* = 8). The randomized, triple blinded, and prospective experimental research was approved on 19 March 2013 by the Ethics Committee in Animal Experimentation (ECAE) of the Puerta de Hierro University Hospital (Madrid, Spain) (ECAE Code: 017/2013). In addition, the study was conducted in accordance with the ethical principles of the ARRIVE guidelines, Royal Decree 1,201/2005 of October 10 (86/609/CEE and ETS 123) on the protection of animals used in experimentation and for other scientific purposes, as well as Council Directive 86/609/EEC of 24 November 1986 and were carried out in accordance with the United Kingdom. Animals (Scientific Procedures) Act 1986, the associated guidelines, and EU Directive 2010/63/EU for animal experiments. The randomized experimental research was performed at the Getafe University Hospital/European University of Madrid (Madrid, Spain) between November 2021 and April 2022. Sample size was determined with a confidence level of 95% (Zα/2 = 1.96), a significance level of 5% (Error *α* = 0.05) and a power of 80% (Error *β* = 0.20; Power = 0.80) using the GRANMO sample size calculator; subsequently, a sample size of 8 dental implants was stablished in each study group.

### 2.2 Conditioning of implant surfaces

The surface of the dental implants (*n* = 16) (Surgimplant IPX, Galimplant, Sarria, Lugo, Spain) were submitted to a dilution of 50 mL tetrahydrofuran (THF) (Uvasol^®^, Madrid, Spain) and 55 mg CEPA for one day at 76°C. Afterwards, the CEPA was activated with a solution of 5 mL distilled water, 175 mL of ethyl-3-(3-dimethylaminopropyl) carboxyamide (EDC) and 54 mg of N-hydroxysulfamide (NHS) for 15 min at room temperature. The stability of the pH was checked (pH 7) using a pH-meter (MP230, Mettler Toledo^®^, Barcelona, Spain) during the full process. EDC activates carboxyl groups and amines by reacting with a carboxyl group to form an O-acylisourea intermediate; however, if it does not react with the amine, it hydrolyzes and regenerates the carboxyl group, thus incorporating the NHS. In the presence of N-hydroxysulfamide, EDC can be used to convert carboxyl groups to amine-reactive N-hydroxysulfamide esters, activating the CEPA with EDC and NHS to react with the amino groups of BMP-7. Once the carboxyl groups were activated, 20 mg of BMP-7 was incubated for 1 h at 37°C.

Finally, the dental implants were exposed to ultrasonic waves to remove impurities, packed in laminar flow cabinets under a sterile atmosphere and sterilized by gamma radiation at 25 KGy. Moreover, the dental implants were blinded to the operator by covering packing.

### 2.3 Experimental procedure

Veterinary assistance was given throughout the study. General anesthesia was induced with intravenous injected propofol 0.2–0.4 mg/kg (Diprivan^®^, AstraZeneca, Cambridge, United Kingdom) using a 20 G needle (BD Microlance^®^, Becton Dickinson, Franklin Lakes, NJ, United States) and epidural anesthesia by injecting bupivacaine (Bupinex^®^, Richmond Vet Pharma, Buenos Aires, Argentina) and fentanyl (Fentanilo^®^, Kilab, Buenos Aires, Argentina). Additionally, local anesthesia was also infiltrated with articaine 4% and 1:200,000 epinephrine (Ultracain^®^, Normon, Madrid, Spain). One N°7 endotracheal tube with a balloon cuff was placed and connected to a circular anesthesia circuit (Leon Plus, Heinen&Löwenstein, Bad Ems, Germany). Multimodal analgesia was used during the study, including medetomidine 0.01 mg/kg (Medetor^®^, Virbac, Carros, France), ketamine 5.0 mg/kg (Ketonal 50^®^, Richmond Vet Pharma, Buenos Aires, Argentina), midazolam 0.2 mg/kg (Dormicum^®^, Roche S.A., Basilea, Switzerland) and atropine 0.02 mg/kg (Atropina^®^, Pharmavet, Bogotá, Colombia). Then, a drilling sequence was performed following the manufacturer´s recommendations using a micromotor (AM-25 E RM, W&H, Bürmoos, Austria) and a contra-angle (WS-75 LG, W&H, Bürmoos, Austria) at 20:1 reduction with profuse saline serum (Vitulia^®^ 0.9%, Barcelona, Spain), after performing an incisión on the tibia. Subsequently, four dental implants (Surgimplant IPX, Galimplant, Sarria, Lugo, Spain) were placed on one tibia of each animal at 40 N torque. Afterwards, multimodal analgesia was administered by a transdermal patch of buprenorphine 0.3 mg/kg (Buprex^®^, Quintiles, Danbury, CT, United States) and meloxicam (Kern Pharma, Madrid, Spain) or buprenorphine 0.05–2 mg/kg (Buprex^®^, Quintiles, Danbury, CT, United States) if neccessary. Moreover, antibiotic therapy of amoxicilin 1.5 g (Clamoxyl^®^, Pfizer, New York, NY, United States) was also administered intramuscularly.

### 2.4 Euthanasia procedure

Animals were randomly (Epidat 4.1, Galicia, Spain) euthanized by an overdose of sodium pentobarbital, under a premedication with Zolacepam-Tiletamina-Medetomidina (Zoletil 5 mg/kg, medetomidine 0.1 mg/kg) administered intramuscularly, four weeks after the surgical intervention.

### 2.5 Histomorphometric analysis

The four tibias were fixed in 10% buffered formalin solution for two weeks to allow histomorphometric processing at the Department of Veterinary Clinical Sciences of University Veterinary Hospital Rof Codina (Lugo, Spain). The dental implants (Surgimplant IPX, Galimplant, Sarria, Lugo, Spain) and bone fragments were then individually extracted with an oscillating autopsy saw (Exakt, Kulzer, Germany) in 16 mm thick sagittal serial sections and dehydrated in semi-liquid alcoholic solutions (80, 96, 100 and 100%) for three days. The samples were embedded in glycolmethacrylate (GMA; 2-hydroxyethyl methacrylate, HEMA, JB-4; JB-4 Plus) (Technovit 7200 VLC, Heraeus Kulzer, Wehrheim, Germany). Finally, the samples were sectioned into 50 µm thick slices (Exakt Aparatebau GMBH, Hamburg, Germany) and stained with the Levai Laczko staining technique and examined with light optical microscopy (BX51, Olympus, Tokyo, Japan) by an experienced pathologist who was unaware of the randomization of the study groups. In addition, images from histological analysis were processed using Adobe Photoshop CS3 (San Jose, CA, United States), digitized (Intuos 4 large, Wacom, Saitama, Japan) and loaded into the Cell Sens Dimensions software, Olympus (Tokyo, Japan) (Balik et al., 2019) in order to evaluate the following variables in an isolated 5 mm × 5 mm area.

- Bone-implant contact (BIC), described as the percentage of the dental implant surface in contact with the surrounding bone.
- Corrected bone-implant contact (BICc), described as the length of bone in contact with the dental implant surface excluding regions non-covered by bone.
- New bone formation (BV/TV), described as the area of new bone formed after placing the dental implant.
- Interthread bone density (BAI/TA), described as the area of threads covered by the surrounding bone.
- Peri-implant bone density (BAP/TA), described as the area of bone that grows along the length of the implant.

All these values were expressed as percentages.

### 2.6 Statistical tests

Statistical analysis of all variables was carried out using SAS 9.4 (SAS Institute Inc., Cary, NC, United State). Descriptive statistics were expressed as means, medians, and standard deviations (SD) for quantitative variables. Comparative analysis was performed by comparing the BIC (%), BICc (%), BV/TV (%), BAI/TA (%) and BAP/TA (%) histomorphometric parameters between the SLA and BMP-7 study groups, using the Student’s t-test and the Mann-Whitney non-parametric test. The statistical significance was set at *p* < 0.05.

## 3 Results

### 3.1 Histomorphometry

**FIGURE 1:**
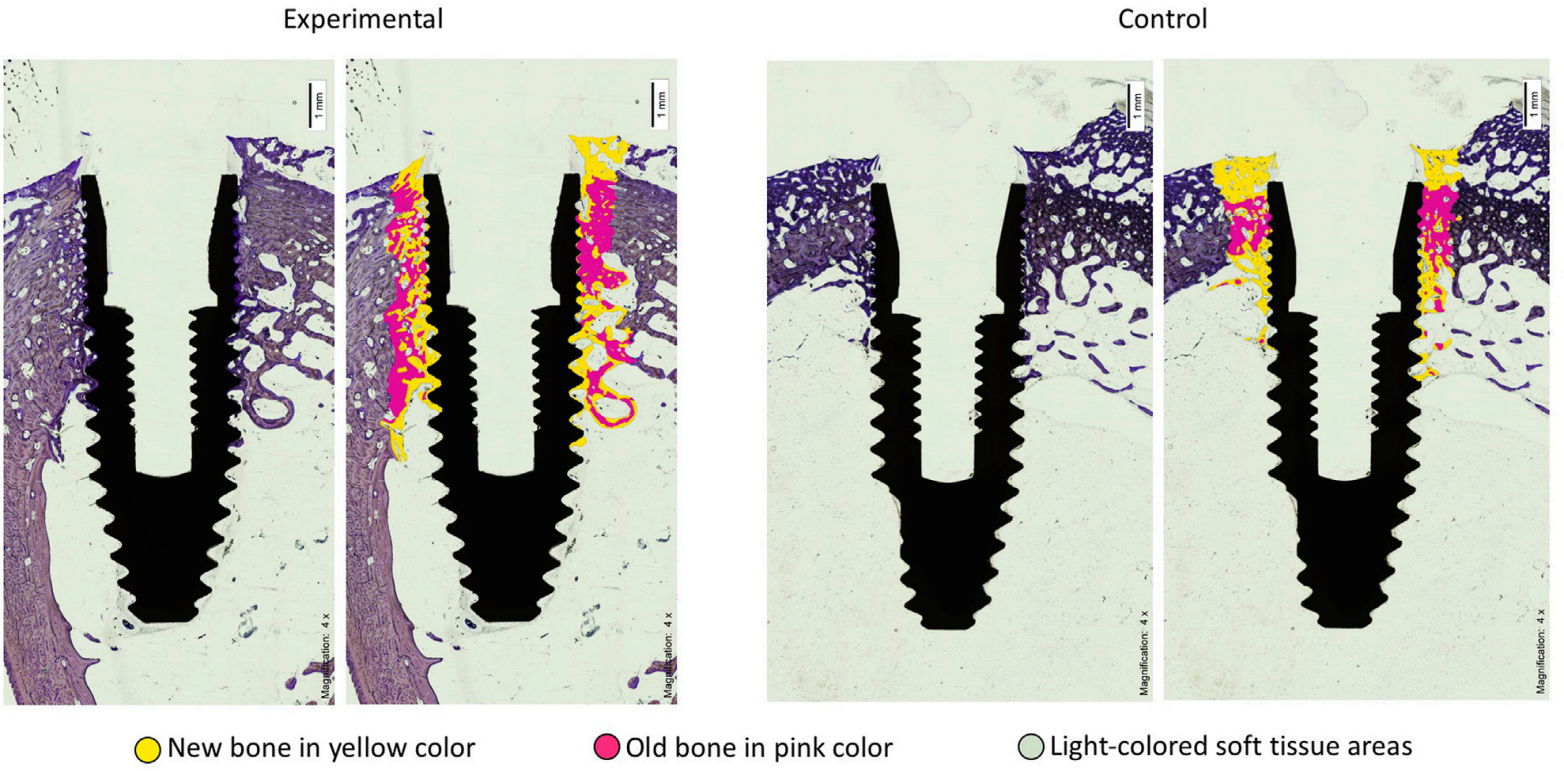
Histomorphometric section images of the BIC of implants in the Acid-BMP-7 (experimental) and SLA (control) groups after four weeks. The yellow color is new bone, more abundant and with greater contact with the implant surface in the experimental group compared to the control group.

Figure 1 shows the longitudinal section of the specimen before being processed by the software for data extraction. The second image of each frame shows, by means of the software, the areas of old bone in pink, the areas of new bone in yellow and the areas of soft tissue in light color. The amount of soft tissue is much more abundant in the control group. The bone-implant contact line is much more extensive in the experimental group.

### 3.2 Statistical results

**FIGURE 2:**
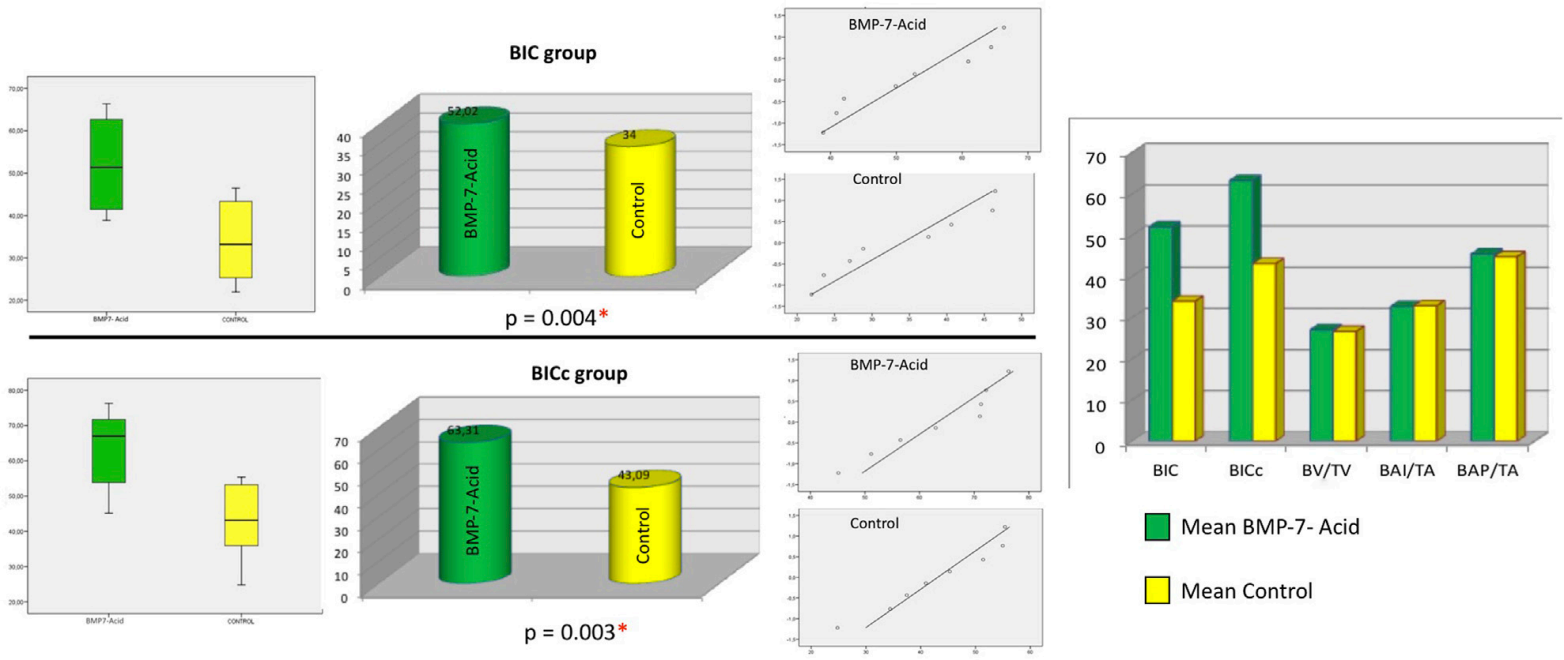
For both BIC and BICc, the box plot shows that the mean value of the BMP7-acid group (test group) was higher than that of the control group. In the Q-Q plots, the points are within the confidence interval and are randomly distributed on both sides of the axis, indicating that the distribution is normal for each group and variable. The bar chart shows the means of the compared groups (statistically significant for the variables BIC and BICc). * Statistical significance.

The means, medians and SD values for BIC (%) histomorphometric parameter of the study groups are displayed in Table 1 and Table 2 and Figure 2.

**TABLE 1:** Descriptive statistics of the BIC (%) histomorphometric parameters.

BIC (%)	*n*	Mean	Median	SD	Minimum	Maximum
SLA	8	34.00^a^	33.17	9.92	21.91	46.47
BMP-7	8	52.02^b^	51.34	10.94	38.88	66.34

a, b different superscripts mean statistically significant differences between groups (*p* < 0.05).

**TABLE 2:** Descriptive statistics of the BICc (%) histomorphometric parameters.

BICc (%)	*n*	Mean	Median	SD	Minimum	Maximum
SLA	8	43.08^a^	43.13	10.76	24.81	55.37
BMP-7	8	63.30^b^	67.00	11.30	45.13	76.27

a, b different superscripts mean statistically significant differences between groups (*p* < 0.05).

Student’s t-test revealed statistically significant differences in histomorphometric parameters BIC and BICc between the experimental and control groups (*p* = 0.004 and *p* = 0.003, respectively). No statistical significance was found between the groups for the other parameters studied; the BAI/TA group obtained values of 32.71 ± 10.87 for the experimental group versus 32.91 ± 7.76 for the control group (*p* = 0.967); the BAP/TA group, 45.47 ± 11.07 for the experimental group versus 44.79 ± 8.67 for the control group (*p* = 0.894) and the BV/TV group, 27.01 ± 6.00 for the experimental group versus 26.63 ± 7.90 for the control group (*p* = 0.951).

## 4 Discussion

Early osseointegration has been shown to be influenced by both the roughness and surface coatings of Ti (Le Guéhennec et al., 2007; Sakka et al., 2012).

Organic coatings on inorganic surfaces, such as Ti, have been widely used to improve their biocompatibility and induce a specific biological response and different *in vivo* investigations demonstrated increased adhesion and proliferation of osteoblastic cells with BMP-2 based coatings (Schliephake et al., 2005; Schliephake et al., 2009).

BMPs act as potent regulators during bone and cartilage formation and repair and both recombinant human BMP-2 and BMP-7 have been approved for clinical use in bone regeneration, fracture healing and spinal fusion (Carreira et al., 2014). Although BMP-2 has been the most studied, certain studies have demonstrated *in vitro* the potential of BMP-7 as a stimulator of bone regeneration (Zhang et al., 2012), it has also been documented that BMP-7 stimulates the maturation of osteoblastic progenitors and induces the differentiation of undifferentiated non-osteogenic cells to osteoblasts (Shen et al., 2010). Busuttil Naudi et al. reported bone regeneration in critical rabbit mandibular defects using beta-tricalcium phosphate (β-TCP) scaffolds and recombinant human bone morphogenetic protein 7 (rhBMP-7) (Busuttil Naudi et al., 2012).

Currently, biomimetic coatings have been developed to induce the formation of a biologically active surface layer of apatite on the implant surface, by developing various techniques capable of creating functional groups (-OH, -COOH, -CH = CH2, etc.). Liu et al. (2002) suggested that pre-deposition of hydroxyapatite (HA) on functionalized self-assembled monolayer surfaces, could be an efficient and fast way to prepare biomimetic apatite coatings on surgical implants. On the other hand, it has been described that the use of acids for peptide anchoring further stimulates the activity of the implant surface, increasing its initial osseointegration; Aresti et al. (2021) demonstrated that the treatment of the surface with CEPA provides functional anchoring groups such as carboxyl groups, and increases the roughness of the dental implant surface, due to the corrosive action of the acid, which causes structural modifications on the surface as a result of the erosive action of the acid, thus showing a more complex surface structure, which induces a greater surface hydrophilicity, which would guarantee a greater affinity for the adhesion and proliferation of osteoblasts to the implant surface. These results would coincide with those found in our study, in which the parameters BIC and BICc were statistically significant between the groups experimental and control.

Other studies reported on the immobilization of BMP-2 after using 11-(hydroxydecyl) phosphonic acid and 12-(carboxydecyl) phosphonic acid to evaluate the reactivity of different surfaces using trifluoroethylamine hydrochloride, since these reactive molecules, can immobilize on the implant surface under the same conditions as a protein, demonstrating a higher covalent immobilization of fluorine molecules in the -COOH group with respect to the -OH group (Adden et al., 2006).

The inducing effect of BMPs on the differentiation of mesenchymal stem cells into osteoblasts has also been demonstrated, which would lead to an improvement in local bone growth, in addition to stimulating the formation of calcium deposits (Hoffmann et al., 2001; Karageorgiou et al., 2004); therefore, these molecules have been extensively studied in dental implantology, in particular BMP-2 (Sykaras et al., 2001; Stadlinger et al., 2008; Wölfle et al., 2014), mainly for their ability to improve osseointegration of dental implants, since BMP-2, BMP-4 and BMP-7 appear to induce comparable levels of bone augmentation (Kirkwood et al., 2003; Leknes et al., 2008; Susin et al., 2010) and in recent years several studies have focused on the role of BMPs in osteoblastic differentiation and their inducing function in collagenous and non-collagenous protein synthesis (Constantinescu-Bercu et al., 2022), however, few studies have focused on analyzing BMP-derived peptides for their osteogenic capacity; Kirkwook et al. (2003), analyzed *in vitro* the minimal bioactive sequences of BMP-7, to evaluate its potential for biomaterial immobilization and its use in controlling osteoblast functions, demonstrating the potential of this molecule to induce osteoblast-specific genes associated with non-collagenous matrix formation and mineralization, along with gene expression, which would suggest some role of BMP-7 in mineralization.

However, there are no uniform results regarding the osteogenic power of BMP-7-functionalized Ti; an *in vitro* study by Togashi et al. (2009) found no effect of the addition of rhBMP-7 to the culture medium on the viability, proliferation, or differentiation of osteoblast-like cells, whereas others have found superior osteogenic power of samples with BMP-7 (Cirano et al., 2014). Zuardi et al. (2022) in a recent study, demonstrated that the effects of BMP-7 on osteogenic differentiation *in vitro*, would be conditioned by the topography of the surface on which the cell cultures are grown, the concentration of the growth factor in the culture medium and the stage of differentiation of the osteoblastic cells. Chen et al. (2013) developed a BMP-7-activated Ti gene delivery system for *in vitro* osteoblast culture and found enhanced differentiation capacity. This demonstrates that strategies for the development of metallic implants functionalized with BMP-7 (and probably with other bioactive molecules) should take into account the release profile during the repair process, depending on the concentration used for functionalization, and the physicochemical characteristics of the implant surface, all with the aim of guaranteeing a specific cellular behavior and/or generating an appropriate tissue phenotype. Nemcakova et al. (2022) in a combined *in vitro* and *in vivo* study found, after 4 and 12 weeks, a more pronounced osteogenic cell maturation and increased mineralization of the extracellular matrix around Ti6Al4V implants functionalized with BMP-7; in contrast, in our study we found no statistical significance in the BAI/TA and BAP/TA parameters. These results coincided with those obtained by Stenport et al. (2003) who, in a study in rabbit tibiae and femurs, found more areas of osteoid-like tissue in implants treated with BMP-7. Susin et al. (2010), Leknes et al. (2008) attribute the results to the concentration of rhBMP-7 used and agree that the observed bone remodeling would be proportional to higher doses of rhBMP-7. Leknes et al. (2008) are in agreement with our study and show robust bone formation after 4 weeks and remodeling of newly formed bone with characteristics similar to those of old bone. As in our study, Hunziker et al. (2021) in an experimental osteoporotic model, found at the third postoperative week, a significantly higher BIC value in the BMP-7 group compared to the Ti metal surface only control group.

Finally, it should be noted that the potential of rhBMP-7 to promote bone formation has been evaluated in a wide variety of large preclinical animal models (Phillips et al., 2006; Hunziker et al., 2021; Schierano et al., 2021) and in our study we used an experimental model with comparable bone anatomy and healing to humans and which has been used extensively in research related to maxillofacial surgery and dental implants. The anatomic and physiologic similarities of the pig to humans in terms of size, physiology, and bone biology have contributed to the successful use of this animal to understand and treat various bone situations and our experimental design was based on previous studies with this animal model, which considered the anatomical characteristics of the tibia to be similar to those of the human mandibular bone in terms of cortical/cancellous ratio and bone quality (Okubo et al., 2002; Mardas et al., 2014).

Our results rejected H_0_ and demonstrated that Ti dental implants exposed to CEPA and coated with BMP-7 exhibit a favorable biological response prone to osseointegration.

Further preclinical studies are needed to investigate the beneficial effects of BMP-7 on bone repair in contact with biomaterials (Senna et al., 2021).

## Data Availability

The original contributions presented in the study are included in the article/Supplementary Material, further inquiries can be directed to the corresponding author.
